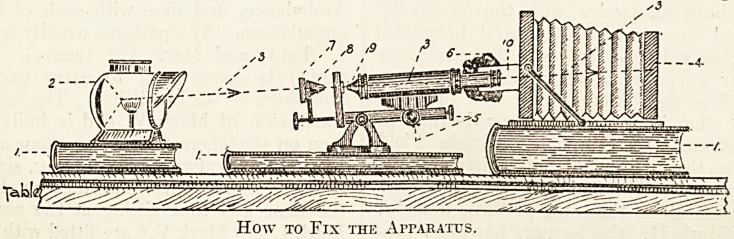# Relaxation and Hobbies: Applied Photography

**Published:** 1912-01-20

**Authors:** 


### RELAXATION AND HOBBIES: Applied Photography.

THE town practitioner has very few real hobbies. The writer, therefore, ventures to think that a short description of his own hobby will not be amiss, especially as it has been successfully piloted through one or two very exacting and extensive club practices. The hobby referred to is that of photography in all its applied branches, including micro- and *x*-ray work, and colour-photography by both methods, through the microscope. The rendering of the colour schemes of pathological sections is so good that the writer hopes that this subject will receive more attention in the future than it has done in the past, for the results are more true to life, and a complete series to illustrate a standard text-book on pathology or bacteriology would make the subject so much more interesting and instructive.

Some of the author's best work has been turned out with the apparatus described below, and has merited inclusion in papers to the Royal Society, and in a recent monograph on the pathology of the blood demonstrating the granular changes in the red and white cells. The microscope used was a Beck fitted with Zeiss ⅔ inch and ⅙ inch objectives and Zeiss eyepieces, having also a Zeiss substage condenser with fitting for colour screens (purchased secondhand for £13). The camera was an old ½-plate one, about which there was no evidence of modernity at all, except the attachment I had made for it. The lens was removed and a brass tube fixed in its place large enough to receive the eyepiece end of the microscope. The source of illumination was an ordinary Lucas' "King of the Road" motor-cycle projector (acetylene), removed from my own motor-cycle.

The camera was placed on the table on a large book. The microscope was then turned till the tube was exactly horizontal, and the mirror removed, and was then put on another book till it was just the right height to fit nicely into the brass tube, already mentioned on the camera. The light was then adjusted to such a height that the optical centre of the beam was in the same straight line as the optical centre of the microscopico-camera combination. The apparatus was now completed by tying a black handkerchief round the moving portions of the apparatus (that is, the brass tube and the eyepiece end of the microscope tube), to exclude any extraneous light. The addition of a Zeiss $\frac{1}{12}$ inch objective (oil immersion) renders the instrument capable of performing work nearly as good as that obtainable by an installation costing nearly £100.

HOW TO FIX THE APPARATUS.

The hobby is not an expensive one, and is really instructive and useful. The camera can also be used, in its spare moments, to take colour-photographs of any interesting skin cases turning up in a large general practice.

## Figures and Tables

**Figure f1:**